# Neutralization of osteopontin attenuates neutrophil migration in sepsis-induced acute lung injury

**DOI:** 10.1186/s13054-015-0782-3

**Published:** 2015-02-26

**Authors:** Yohei Hirano, Monowar Aziz, Weng-Lang Yang, Zhimin Wang, Mian Zhou, Mahendar Ochani, Adam Khader, Ping Wang

**Affiliations:** Department of Surgery, Hofstra North Shore-LIJ School of Medicine and Center for Translational Research, The Feinstein Institute for Medical Research, 350 Community Drive, Manhasset, NY 11030 USA; Department of Emergency and Critical Care Medicine, Juntendo University, Urayasu Hospital, 2-1-1 Tomioka, Urayasu, Chiba 279-0021 Japan

## Abstract

**Introduction:**

Sepsis refers to severe systemic inflammation leading to acute lung injury (ALI) and death. Introducing novel therapies can reduce the mortality in ALI. Osteopontin (OPN), a secretory glycoprotein produced by immune reactive cells, plays a deleterious role in various inflammatory diseases. However, its role in ALI caused by sepsis remains unexplored. We hypothesize that treatment with an OPN-neutralizing antibody (anti-OPN Ab) protects mice against ALI during sepsis.

**Methods:**

Sepsis was induced in 8-week-old male C57BL/6 mice by cecal ligation and puncture (CLP). Anti-OPN Ab or non-immunized IgG as control, at a dose of 50 μg/mouse, was intravenously injected at the time of CLP. After 20 hours, the expression of OPN and proinflammatory cytokines in tissues and plasma was examined by real-time PCR, Western blot, and ELISA. Plasma levels of alanine aminotransferase (ALT), aspartate aminotransferase (AST) and lactate dehydrogenase (LDH) and the lung myeloperoxidase (MPO) levels were determined by colorimetric assays. Lung damage and neutrophil infiltrations were determined by histological H&E and Gr-1 staining, respectively. The effect of recombinant mouse OPN (rmOPN) on human neutrophil-like cell (HL-60) migration was performed by Boyden chamber assays and the involvement of intracellular signaling molecules in HL-60 cells was revealed by Western blot.

**Results:**

After 20 hours of sepsis, mRNA and protein levels of OPN were significantly induced in lungs, spleen, and plasma. Treatment with an anti-OPN Ab in septic mice significantly reduced the plasma levels of ALT, AST, and LDH, and the proinflammatory cytokines IL-6, IL-1β and the chemokine MIP-2, compared with the vehicle group. Similarly, the lung mRNA and protein expressions of proinflammatory cytokines and chemokine were greatly reduced in anti-OPN Ab-treated animals. The lung histological architecture, MPO and neutrophil infiltration were significantly improved in anti-OPN Ab-treated mice compared with the vehicle animals. Treatment of rmOPN in HL-60 cells significantly increased their migration, *in vitro*. The neutrophils treated with rmOPN remarkably increased the levels of phospho focal adhesion kinase (pFAK), phospho extracellular signal-regulated kinase (pERK) and phospho p38.

**Conclusions:**

Our findings clearly demonstrate the beneficial outcomes of anti-OPN Ab treatment in protecting against ALI, implicating a novel therapeutic strategy in sepsis.

## Introduction

Sepsis is a severe systemic inflammation that occurs due to an abnormal host response against invading pathogens. Septic patients suffer from a ‘cytokine storm’ as a result of an exaggerated production of proinflammatory cytokines, chemokines and other inflammatory mediators [1]. Severe sepsis, complicated by acute organ dysfunction, is recorded in 10% of all intensive care unit (ICU) admissions and represents the most common cause of death among hospitalized patients in the USA [2,3]. Acute lung injury (ALI) or its more severe condition, acute respiratory distress syndrome (ARDS) is the primary complication in sepsis during the sequential development of multiple organ dysfunction (MOF) [4]. Despite tremendous clinical and scientific effort in this field, ALI/ARDS in sepsis remain as considerable challenges to critical care medicine, causing approximately 74,500 deaths per year [5]. Hence, the development of novel therapeutic strategies for ALI/ARDS is invaluable for critical care patients.

In sepsis, activated neutrophils transmigrate and infiltrate into the lungs where they release a large number of proteolytic enzymes, such as myeloperoxidase (MPO), and reactive oxygen species (ROS) including hydrogen peroxide and superoxide for elimination of invaded pathogens [6,7]. However, overwhelming migration of activated neutrophils into the lungs can lead to a deleterious function by causing endothelial cell damage and unrestrained inflammation [8,9]. In ARDS patients, the intensity of neutrophil infiltrations correlates with the severity of lung injury caused by neutrophil-derived proteolytic enzymes and proinflammatory mediators in the bronchoalveolar lavage [10]. Thus, it implies that the regulation of neutrophil infiltration into the lungs could be an effective therapeutic approach in septic patients with ALI/ARDS.

Osteopontin (OPN) also known as bone sialoprotein I (BSP-I), early T lymphocyte activation (ETA-1), or secreted phosphoprotein 1 (SPP-1), is shown to be multifunctional and implicated in the pathogenesis of a variety of diseases [11-17]. In the immune system, OPN is synthesized by a variety of immune cells including activated T cells, natural killer (NK) cells, dendritic cells (DC), and macrophages [18,19]. Through interactions with several integrins or CD44, OPN mediates diverse cellular functions such as adhesion, migration, and survival of several different cell types, including regulating and propagating inflammatory responses of macrophages, T cells, and DC [11]. The existence of variant forms of OPN as a secreted (sOPN) and intracellular (iOPN) protein and its modification through posttranslational events and proteolytic cleavage explains its broad range of functions [20]. Nascent OPN protein undergoes posttranslational modifications including serine/threonine phosphorylation, sulfation, glycosylation and transglutamination [11]. In addition, OPN is also a substrate for thrombin and the matrix metalloproteinases, (MMP)-3, -7, -2, -9 and -12 to generate its different cleaved fragments [11,21]. In human, OPN contains three cleavage sites for MMPs, that is Gly_166_-Leu_167_, Ala_201_-Tyr_202_, and Asp_210_-Leu_211_ [11]. Agnihotri *et al.* showed that cleavage of the full-length OPN by MMP-3 and -7 at Gly_166_-Leu_167_ generates 40- and 32-kiloDalton (kDa) N- and C-terminal fragments, respectively. The resultant 32-kDa C-terminal fragment is further cleaved by thrombin at Arg_168_-Ser_169_ to form a 25-kDa fragment [22]. The thrombin-cleaved N-terminal fragment containing a RGD and SVVYGLR sequences is capable of binding to several integrins, such as α_v_β_3_, α_v_β_5_, α_9_β_1_, α_4_β_1_ and others to promote biological functions [11]. On the other hand, the C-terminal fragments transduce intracellular signals by binding to CD44 [23]. Compared with chronic inflammatory diseases, fewer reports focus on acute inflammatory diseases or infection on OPN function [24-27]. It is therefore crucial to delineate the pathophysiological role of OPN in sepsis-induced ALI, and also becomes necessary to know whether or not neutralization of OPN can ameliorate this acute inflammatory disease condition. Within the diverse functions, OPN can act as a chemoattractant for T cells, monocytes/macrophage and neutrophils [28,29]. Considering the deleterious function of exaggerated infiltration of neutrophils in lungs to cause sepsis-induced ALI, we hypothesize that the blockage of OPN by its neutralizing antibody (Ab) may effectively reduce neutrophil migration into the lungs by modulating intracellular signaling molecules required for cell migration, ultimately attenuating sepsis-induced ALI.

## Materials and methods

### Animal model of sepsis

Eight-week-old male C57BL/6 mice purchased from Taconic Biosciences (Albany, NY, USA) were housed in a temperature-controlled room on a 12 h light/dark cycle and fed a standard laboratory diet. Sepsis was induced in mice by cecal ligation and puncture (CLP). Mice were anesthetized by isoflurane inhalation, and the abdomen was shaved and wiped with 10% povidone iodine (PI). A 1-cm abdominal incision was performed to expose the cecum. The cecum was tightly ligated with a 4-0 silk suture 0.5 to 0.75 cm away from the tip and punctured twice between the tip and the ligation with a 22-gauge needle to eject a small amount of feces from the perforation sites by gentle squeezing. The cecum was returned to the abdominal cavity and the laparotomy site was closed with a 6-0 silk suture in two layers. The sham animals underwent the same procedure with the exception of the cecum neither ligated nor punctured. Animals were resuscitated with 1 ml of normal saline subcutaneously. At 20 h after operation, mice were anesthetized and blood, spleen and lung samples were collected. Blood samples were centrifuged at 3,000 g for 10 min to collect plasma. The plasma and tissue samples were frozen immediately in liquid nitrogen and stored at -80°C until analysis. A section of lung tissue was preserved in formalin for histopathological analysis. All experiments were performed in accordance with the guidelines for the use of experimental animals by the National Institutes of Health (Bethesda, MD, USA) and were approved by the Institutional Animal Care and Use Committee (IACUC) at the Feinstein Institute for Medical Research.

### Administration of neutralizing OPN antibody

Mouse affinity purified polyclonal Ab, anti-OPN Ab purchased from R&D Systems, Minneapolis, MN, USA; Catalog No.: AF808, was used for neutralization of OPN *in vivo.* According to the literature, the polyclonal anti-OPN Ab is able to neutralize recombinant OPN-mediated cell adhesion at a ND_50_ of 1 to 3 μg/ml, *in vitro.* In addition, recent studies also demonstrated its *in vivo* neutralizing function against endogenous OPN when injected into experimental animals [30,31]. Immediately after CLP, a small incision on the neck was performed to expose the internal jugular vein. Phosphate-buffered saline (PBS) or mouse anti-OPN Ab at a dose of 50 μg/mouse in 100 μl volumes was delivered by bolus injection through the jugular vein to serve as vehicle and treatment groups, respectively. In the same way, normal goat immunoglobulin G (IgG) (R&D Systems; Catalog No.: AB-108-C) at a dose of 50 μg/mouse in 100 μl volumes was injected in septic animals to serve as the non-immunized IgG control for anti-OPN Ab. The proximal and distal ends of the injected jugular vein were tightly ligated with 5-0 silk suture to prevent unwanted bleeding caused by injecting with anti-OPN Ab using 29G × 1/2″ U-100 insulin syringe (Terumo Medical Corporation, Elkton, MD, USA). Ligation of one of the paired veins did not interrupt the blood flow from the head back to the heart. The wound was closed with one interrupted 6-0 silk suture.

### Measurement of organ injury markers

Plasma levels of aspartate aminotransferase (AST), alanine aminotransferase (ALT) and lactate dehydrogenase (LDH) were measured using commercial assay kits (Pointe Scientific, Lincoln Park, MI, USA) according to the manufacturer’s instructions.

### Measurement of proinflammatory cytokines and chemokine

Proinflammatory cytokines, interleukin 6 (IL-6) and IL-1β in plasma and lung tissues were quantified by using the mouse enzyme-linked immunosorbent assay (ELISA) kits (BD Biosciences, Franklin Lakes, NJ, USA). Macrophage inflammatory protein (MIP)-2 in plasma and lung tissues was measured by using the mouse ELISA kit (R&D Systems).

### Histological examination and immunostaining

The lung tissue was fixed in 10% formalin and then embedded in paraffin. Later, the tissue blocks were cut into 5-μm sections, placed onto glass slides and stained with hematoxylin and eosin (H&E), dehydrated, and mounted. Morphologic examinations in these tissues were evaluated by light microscopy in a blinded fashion. To examine the extent of lung injury, we considered its five pathological features, such as (i) presence of exudates, (ii) hyperemia/congestion, (iii) intra-alveolar hemorrhage/debris, (iv) cellular infiltration, and (v) cellular hyperplasia. The severity of each of these pathological features was evaluated by a score indicating 0 as absent/none, 1 as mild, 2 as to show moderate, and finally 3 for severe injury. Compilations of these values obtained from individual pathological features represent the lung injury score [7,32].

Gr-1 is a 21- to 25-kDa myeloid differentiation protein and also known as Ly-6G/Ly-6C. This myeloid differentiation antigen is a glycosylphosphatidylinositol (GPI)-linked protein expressed on granulocytes and macrophages. In the bone marrow, expression levels of Gr-1 directly correlate with granulocyte differentiation and maturation [33]. To examine neutrophil infiltration in lungs we performed immunohistochemistry using anti-Gr-1 Ab (BioLegend, San Diego, CA, USA; Catalog No.: 108413) as described previously [7]. In brief, 10% formalin-fixed, paraffin-embedded lung tissues were dewaxed in xylene and rehydrated in a graded series of ethanol. The slides were heated in 0.92% citric acid buffer (Vector Laboratories, Burlingame, CA) at 95°C for 30 min. After cooling to room temperature, the slides were incubated with 2% H_2_O_2_ in 60% methanol and blocked in 2% normal rabbit serum/Tris-buffered saline. Anti-Gr-1 antibody (BioLegend) was then applied and incubated overnight. Vectastain ABC reagent and DAB kit (Vector Laboratories) were used to detect the immunohistochemical reaction. Slides were counterstained with 4′, 6-diamidino-2-phenylindole and examined under a phase contrast light microscope (Eclipse Ti-S; Nikon, Melville, NY, USA). Gr-1-positive staining cells were counted in 10 visual fields/section at × 200 magnification, and averaged number was calculated.

### Myeloperoxidase activity assay

Lung tissues were homogenized in potassium phosphate buffer containing 0.5% hexadecyl trimethyl ammonium bromide by sonication. After the samples were centrifuged, the supernatant was diluted in reaction solution containing o-dianisidine hydrochloride and H_2_O_2_ in PBS. The rate of change in optical density (OD) for 1 min was measured at 460 nm to calculate MPO activity.

### Quantitative real-time RT-PCR analysis

Total RNA was extracted from lung tissues by using TRIzol (Invitrogen, Carlsbad, CA, USA) and was reverse-transcribed into cDNA using reverse transcriptase (Applied Biosystems, Foster City, CA, USA). A polymerase chain reaction (PCR) was carried out in 25 μl of final volume containing 0.1 μM of each forward and reverse primer, cDNA and 12.5 μl SYBR Green PCR Master Mix (Life Technologies, Grand Island, NY, USA). Amplification was conducted using an Applied Biosystems 7300 real-time PCR machine under the thermal profile of 50°C for 2 min, 95°C for 10 min followed by 45 cycles of 95°C for 15 seconds and 60°C for 1 min. For relative quantization, 2^-ddCt^ method normalized to mouse β-actin mRNA was used. Relative expression of mRNA was expressed as the fold change in comparison with the sham tissues. The primers used for this study are: OPN (NM_001204201) Forward: TCTGATGAGACCGTCACTGC, Reverse: AGGTCCTCATCTGTGGCATC; IL-6 (NM_031168) Forward: CCGGAGAGGAGACTTCAC AG, Reverse: GGAAATTGGGGTAGGAAGGA; MIP-2 (NM_009140) Forward: CCCTGG TTCAGAAAATCATCCA, R: GCTCCTCCTTTCCAGGTCAGT; IL-1β (NM_008361) F: CAGGATGAGGACATGAGCACC, R: CTCTGCAGACTCAAACTCCAC; β-Actin (NM_00 7393): F-CGTGAAAAGATGACCCAGATCA, R-TGGTACGACCAGAGGCATACAG.

### Migration assay

Human promyelocytic HL-60 cells were obtained from the American Type Culture Collection (Manassas, VA, USA) and cultured in RPMI medium (Invitrogen) containing 10% fetal bovine serum (FBS), glutamine, penicillin and streptomycin. HL-60 cells were differentiated into neutrophil-like cells (dHL-60) by adding dimethyl sulfoxide (DMSO) at (12.7 μl/ml per million cells) for 5 days. The differentiated HL-60 cells (1.5 × 10^6^ cell/ml) were pretreated with PBS (DMSO), or extracellular signal-regulated protein kinase (ERK) inhibitor (PD98059, 50 μM), or p38 inhibitor (SB203580, 50 μM) for 1 h. The migration assays were performed in a modified 24-well (3.0-μm pore) Boyden chamber using falcon cell culture inserts (BD Biosciences). Recombinant mouse osteopontin (rmOPN) purchased from R&D Systems (Catalog No.: 441-OP) was used as a chemoattractant for dHL-60 cell migration. rmOPN was prepared by cloning the cDNA encoding Leu17 to Asn294 (Glu99Gly) of OPN transcript without its signal peptide sequence (Accession No.: Q547B5) into a C-terminal 6-His tag expression vector. The expression and purification of rmOPN protein were carried out in a mouse myeloma cell line, NS0. The cells were plated in the upper well, and medium containing PBS or rmOPN at a dose of 10 μg/ml was placed in the lower well as a chemotactic stimulus. After 2 h, the upper surface of the filter was washed and swabbed with cotton to remove non-migratory cells. Migrated cells were fixed with 10% formalin. PI was used for staining the migrated cells and observed under a fluorescence microscope. Cell number was counted in five random microscopic fields per well. In order to examine the role of rmOPN for primary neutrophil migration, mice bone marrow neutrophils were isolated as described previously [6] and the migration assay was performed by following the above method used for HL-60 cell migration.

### Intratracheal administration of rmOPN and assessment of neutrophil migration in lungs

Male (25 to 30 g) age-matched C57BL/6 J (Taconic Biosciences) mice were anesthetized with isoflurane and the trachea was surgically exposed, lifted, and then instilled with either 40 μl of sterile PBS or rmOPN at a dose of 2.5 μg/mouse using 29G × 1/2″ U-100 Insulin Syringe (Terumo Medical Corporation). The wound was closed with 6-0 silk suture. After 20 h of rmOPN administration, lung tissues were removed and cut into 300 mm pieces, and digested in RPMI 1640 containing 10% FBS, 100 U penicillin/streptomycin, 20 mM L-glutamine, 100 U/ml collagenase type 1 (Worthington Biochemical Corp., Lakewood, NJ, USA) and 20 U/ml DNase 1 (Roche Diagnostics, Mannheim, Germany) for 60 min in a 37°C water bath with shaking. Any remaining intact tissue was disrupted by passage through a 21G needle. The digested tissue contents were then passed through a 70 μm Nylon mesh (BD Biosciences), centrifuged and washed. A total of 1 × 10^6^ cells were stained with APC-labeled anti-mouse Gr-1 Ab (BioLegend) and subjected for flow cytometry analysis.

### Western blot analysis

Spleen and lung tissues and dHL-60 cells (1.5 × 10^6^ cells) treated with 2 μg/ml of rmOPN for 90 min were homogenized in lysis buffer (10 mM Tris-HCl, pH 7.5, 120 mM NaCl, 1% NP-40, 1% sodium deoxycholate and 0.1% sodium dodecyl sulfate) containing a protease inhibitor cocktail (Roche Diagnostics). Protein concentration was determined by Bio-Rad Laboratories (Hercules, CA, USA) protein assay reagent. Total lysates and plasma were fractionated on Bis-Tris gels (4% to 12%) and transferred to nitrocellulose membrane. The membranes were then blocked with 5% nonfat dry milk in Tris-buffered saline with Tween-20 (TBST) and incubated overnight at 4°C with the primary antibodies as obtained from respective vendors: anti-OPN Ab (Santa Cruz Biotechnology, Santa Cruz, CA, USA), anti-phospho-focal adhesion kinase (FAK) (Tyr397) Ab, anti-FAK Ab, anti-phospho-p44/42 mitogen-activated protein (MAP) kinase (pERK) (Thr202/Tyr204) Ab, anti-p44/42 MAP kinase (ERK) Ab, anti-phospho-p38 MAP kinase (Thr180/Tyr182) Ab, and anti-p38 MAP kinase Ab (Cell Signaling Technology, Beverly, MA, USA), and anti-β-actin Ab from Sigma-Aldrich (St Louis, MO, USA).

### Statistical analysis

Data are expressed as mean ± standard error of the mean (SEM) and analyzed using Sigma Plot11 graphing and statistical analysis software (Systat Software Inc., San Jose, CA, USA). Multiple groups were compared by one-way analysis of variance (ANOVA) using the Student-Newman-Keuls (SNK) test. Student’s *t* test was used for two-group analysis. Differences in values were considered significant if *P* <0.05.

## Results

### OPN expression is upregulated in the lungs, spleen and plasma after sepsis

**Figure 1:**
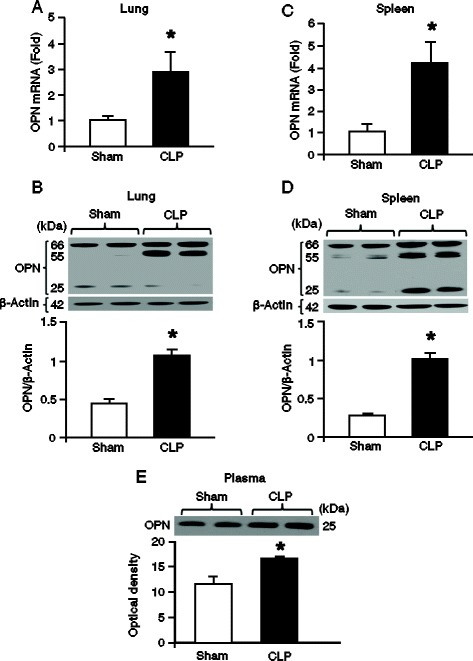
**Expression of OPN in lungs, spleen and plasma after sepsis in mice** ***.*** Lungs, spleen and blood samples were harvested at 20 h after CLP or sham operation. Lung expression of OPN at its **(A)** RNA and **(B)** protein levels was measured by using real-time PCR and Western blot, respectively. OPN expression in the spleen tissues was determined at its **(C)** RNA and **(D)** protein levels. OPN expression in each sample was normalized to β-actin expression and the value of sham group was designated as one for comparison. **(E)** OPN expression in 3.0 μL of plasma from sham and CLP animals was determined by Western blot. Each blot was quantified by densitometry analysis. Representative blots against OPN and β-actin are shown. Data are expressed as means ± SEM (n = 5 mice/group) and compared by Student’s *t* test (^*^ *P* <0.05 vs. shams). CLP, cecal ligation and puncture; OPN, osteopontin; PCR, polymerase chain reaction; SEM, standard error of the mean.

To assess the alteration of OPN expression after 20 h of CLP, the lung and spleen tissues of sham and CLP mice were examined by Western blot and quantitative PCR analysis. In the lung tissues, the RNA expression of OPN in the CLP group was significantly upregulated by 1.9-fold compared to the sham mice (Figure 1A). While analyzing the protein expression by Western blot, we detected three bands with the molecular weights of about 65-, 55-, and 25-kDa, respectively, which represented different cleaved fragments of OPN (Figure 1B). Summing up the densitometry values of all the OPN fragments in the lung tissues of each group showed significant upregulation of its total contents in CLP animals as compared to the sham animals (Figure 1B). Similarly, in spleen, the RNA and total protein expression of OPN in the CLP group were significantly upregulated by 3.2- and 2.0-fold in CLP animals than that of sham group, respectively (Figures 1C,D). In contrast, instead of multiple bands the plasma samples showed 25-kDa OPN form alone. The reason for not getting the 65- and 55-kDa OPN bands in plasma samples could be due to the abundance of albumin (molecular weight: 66 kDa) in the plasma, which hampered the detection of other posttranslational modified forms of OPN in plasma by Western blot. As shown in Figure 1E, the 25-kDa OPN fragment was elevated in the plasma from the CLP mice as compared to the sham group.

### OPN neutralization decreases organ injury and systemic inflammatory responses after sepsis

**Figure 2:**
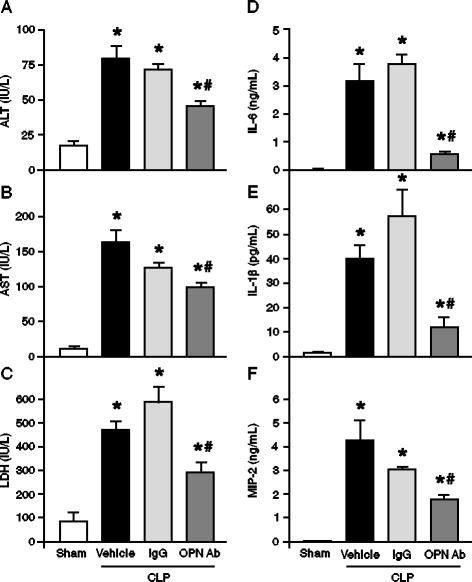
**Effect of anti-OPN Ab treatment on plasma levels of organ injury markers and proinflammatory cytokines and chemokine in CLP animals** ***.*** Sepsis was induced in mice by CLP and anti-OPN Ab or non-immunized IgG control at a dose of 50 μg/mice in 100 μl volumes was injected through the jugular vein. In the vehicle group, 100 μl of PBS was injected in CLP mice via the jugular vein. Blood samples were drawn by cardiac puncture at 20 h of sham-operated, vehicle and anti-OPN Ab-treated mice for measuring **(A)** ALT, **(B)** AST and **(C)** LDH. Similarly, the blood samples collected at 20 h after CLP were measured for **(D)** IL-6, **(E)** IL-1β and **(F)** MIP-2 by ELISA. Data are expressed as means ± SEM (n = 5 mice/group) and compared by one-way ANOVA and SNK method (^*^ *P* <0.05 vs. shams; ^#^ *P* <0.05 vs. vehicle). Ab, antibody; ALT, alanine aminotransferase; ANOVA, analysis of variance; AST, aspartate aminotransferase; CLP, cecal ligation and puncture; ELISA, enzyme-linked immunosorbent assay; IgG, immunoglobulin G; IL, interleukin; LDH, lactate dehydrogenase; MIP-2, macrophage inflammatory protein 2; OPN, osteopontin; PBS, phosphate-buffered saline; SEM, standard error of the mean; SNK, Student-Newman-Keuls.

Clinical markers such as AST, ALT, and LDH in blood were measured at 20 h after CLP in mice to assess the extent and severity of organ injury. Compared with the sham group, the levels of AST, ALT and LDH were significantly elevated in the vehicle group, as well as in the non-immunized IgG control-treated animals (Figures 2A-C). In contrast, the treatment with anti-OPN Ab significantly reduced the levels of these injury markers by 38%, 42%, and 45%, respectively, as compared with the vehicle group (Figures 2A-C). We have also examined the effect of anti-OPN Ab treatment on systemic levels of proinflammatory cytokines and chemokine. The plasma levels of IL-6, IL-1β and MIP-2 were significantly increased in the vehicle group as compared with the sham group, whereas treatment with anti-OPN Ab significantly decreased the plasma levels of IL-6, IL-1β and MIP-2 by 88%, 73% and 60%, respectively, as compared with the vehicle animals (Figure 2D-F). Conversely, the animals injected with non-immunized IgG control did not show any notable reduction in the levels of plasma cytokines and chemokine as compared with the vehicle group. Since we could not find any noticeable difference of plasma injury as well as inflammation markers between vehicle and non-immunized IgG control groups, it is therefore logical that the parameters in tissues after treatment with IgG control might not differ from the vehicle group.

### OPN neutralization inhibits cytokines and chemokine expression in the lungs after sepsis

**Figure 3:**
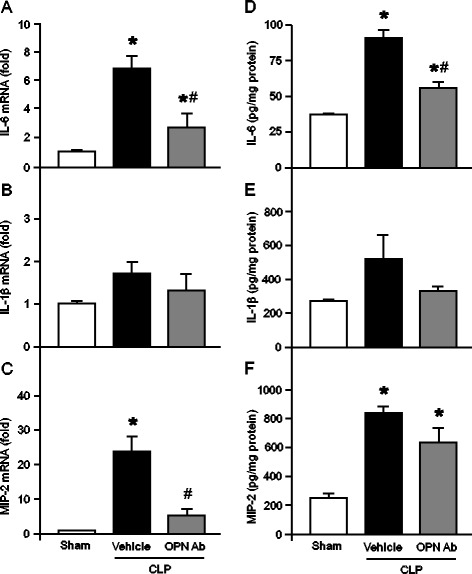
**Effect of anti-OPN Ab on the expression of proinflammatory cytokines and chemokine in the lungs after CLP** ***.*** Mice were subjected to CLP and 100 μl of anti-OPN Ab (50 μg/mouse) or PBS as vehicle was injected through the jugular vein. Lung tissues were collected after 20 h from sham-operated, vehicle, and anti-OPN Ab-treated mice. The tissue expression of **(A)** IL-6, **(B)** IL-1β and **(C)** MIP-2 was determined by real-time PCR. Gene expression was normalized to β-actin. The sham expression level was designated as one for comparison. Similarly, 50 μg of total protein extracted from the lung tissues were examined for **(D)** IL-6, **(E)** IL-1β and **(F)** MIP-2 analysis by ELISA. Finally, the results are expressed as per mg of proteins. Data are represented as means ± SEM (n = 5 mice/group) and compared by one-way ANOVA and SNK method (^*^ *P* <0.05 vs. sham; ^#^ *P* <0.05 vs. vehicle). Ab, antibody; ANOVA, analysis of variance; CLP, cecal ligation and puncture; ELISA, enzyme-linked immunosorbent assay; IL, interleukin; MIP-2, macrophage inflammatory protein 2; OPN, osteopontin; PBS, phosphate-buffered saline; PCR, polymerase chain reaction; SEM, standard error of the mean; SNK, Student-Newman-Keuls.

Apart from the blood, we have also investigated the expressions of cytokines and chemokine in the lung tissues at their RNA and protein levels by real-time PCR and ELISA, respectively. Both the RNA and protein contents of IL-6 in the lung tissues were significantly increased in the vehicle group as compared with the sham group (Figure 3A,D). However, the treatment with anti-OPN-Ab significantly inhibited its RNA and protein levels by 36% and 60%, respectively, as compared with the vehicle mice (Figure 3A,D). The expression levels of IL-1β in both RNA and proteins in the lung tissues were markedly upregulated in the vehicle group as compared with the sham group. Although not significant there was a clear trend in downregulating its RNA and protein levels by 34% and 28%, respectively, in anti-OPN Ab-treated animals than the vehicle group (Figure 3B,E). The RNA expression of MIP-2 in the lung tissues was significantly increased in the vehicle group as compared with the sham group and it was dramatically decreased in anti-OPN-Ab-treated animals by 79% than the vehicle mice (Figure 3C). Although not significant, MIP-2 protein expression in the lungs was found to be notably decreased by 21% after anti-OPN-Ab treatment in septic mice as compared with the vehicle-treated animals (Figure 3F).

### OPN neutralization protects against sepsis-induced ALI in mice

**Figure 4:**
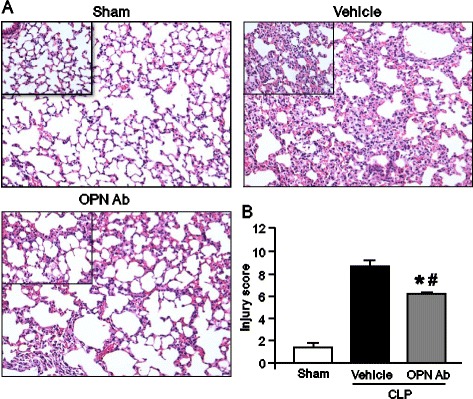
**Evaluation of lung histology in mice after CLP** ***.*** Lung tissues were harvested after 20 h from sham-operated, vehicle and anti-OPN Ab-treated mice and stained with H&E. Slides were observed under light microscopy at × 200 original magnifications (inset: ×400 original magnification). **(A)** Representative images for sham, vehicle, and anti-OPN Ab treatment groups are shown. **(B)** Histological injury scores of the lungs in different groups were quantified as described in the Materials and Methods. Data are expressed as means ± SEM (n = 5 mice/group) and compared by one-way ANOVA and SNK method (^*^ *P* <0.05 vs. shams; ^#^ *P* <0.05 vs. vehicles). Ab, antibody; ANOVA, analysis of variance; CLP, cecal ligation and puncture; H&E, hematoxylin and eosin; OPN, osteopontin; SEM, standard error of the mean; SNK, Student-Newman-Keuls.

To clarify the effect of neutralizing OPN on the ALI caused by CLP, lung tissue histology was performed and the severity of injury was graded with an established scoring system as described in the Materials and Methods. Representative histological architectures of the sham, vehicle, and anti-OPN-Ab-treated mice are shown in Figure 4A. The lung tissues in the vehicle group presented substantial morphological changes including edema, hemorrhage, alveolar collapse, and inflammatory cell infiltrations as compared with the sham group. In contrast, the treatment with anti-OPN-Ab dramatically reduced the microscopic deterioration in comparison with the vehicle group. As quantified in Figure 4B, the lung tissue in the vehicle group exhibited a significant increase in the histological injury score as compared with the sham group, whereas the treatment with anti-OPN Ab significantly improved the lung histological injury score as compared with vehicle group.

### OPN neutralization reduces neutrophil infiltration and MPO activity in the lungs after sepsis

**Figure 5:**
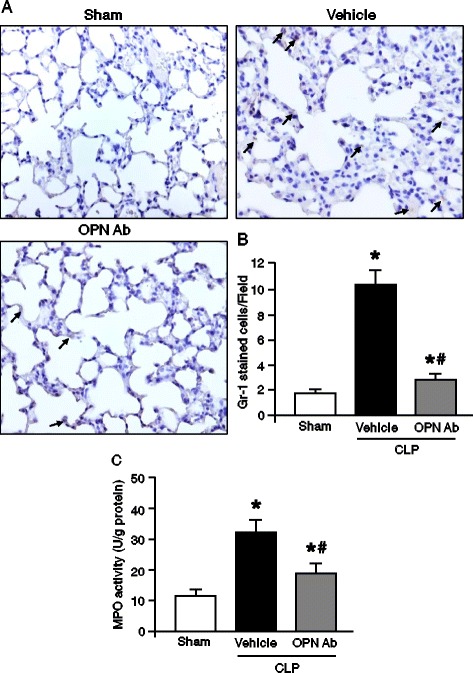
**Assessment of neutrophil infiltration into the lungs after CLP** ***.*** Lung tissues were collected at 20 h after sham-operation, vehicle and anti-OPN Ab treatment in mice. **(A)** Representative images of the lung tissue sections with immunostaining against Gr-1 at × 200 original magnification are shown. Arrows demarcate examples of areas of staining Gr-1-positive cells. **(B)** A graphical representation of Gr-1-positive cells averaged over 10 microscopic fields per animal lung tissues. **(C)** Myeloperoxidase (MPO) activities in lung tissues were determined spectrophotometrically. Data are expressed as means ± SEM (n = 5 mice/group) and compared by one-way ANOVA and SNK method (^*^ *P* <0.05 vs. shams; ^#^ *P* <0.05 vs. vehicles). Ab, antibody; ANOVA, analysis of variance; CLP, cecal ligation and puncture; OPN, osteopontin; SEM, standard error of the mean; SNK, Student-Newman-Keuls.

ALI is directly correlated with the number of infiltrated neutrophils in the lungs. We therefore performed Gr-1 staining, a surface marker of activated neutrophils in the lungs, after 20 h of CLP and the representative pictures of immunohistochemistry in the sham, vehicle, and anti-OPN-Ab-treated mice are shown in Figure 5A. The number of Gr-1-positive cells in the lung tissues was significantly increased in the vehicle group as compared with the sham group. In contrast, there were significantly lower numbers of Gr-1-positive cells in the OPN-Ab-treated group than the vehicle group mice (Figures 5A,B). Furthermore, the tissue damage in ALI is also linked with the MPO activity within the lung. The MPO activity in the lung tissue harvested at 20 h after CLP was significantly increased in the vehicle group as compared with the sham animals, whereas injecting anti-OPN Ab significantly inhibited its activity by 37% in comparison with the vehicle group (Figure 5C).

### Administration of rmOPN intratracheally induces neutrophil migration in lungs

**Figure 6:**
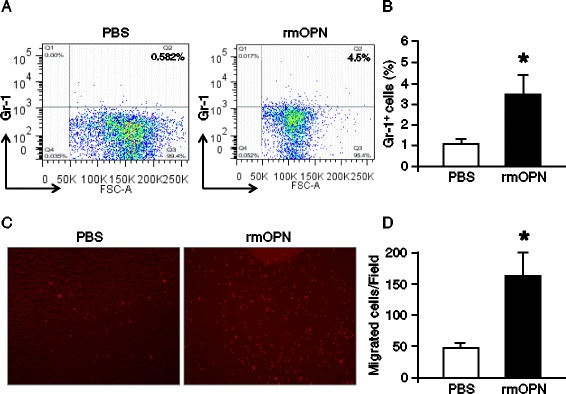
**rmOPN-mediated neutrophil migration** ***in vivo*** **and** ***in vitro.*** C57BL/6 mice were injected with rmOPN at a dose of 2.5 μg/mice, intratracheally. After 20 h, cells from the lung tissues were isolated and stained with APC-anti-Gr-1 Ab and then subjected to flow cytometry. **(A)** Representative dot blots indicating the percentages of Gr-1-positive cells are shown. **(B)** The mean percentages of Gr-1-positive cells obtained from PBS- and rmOPN-injected mice are shown. Data are expressed as means ± SEM (n = 4 mice/group) and compared by Student’s *t* test (^*^ *P* <0.05 vs. PBS). **(C)** A total of 5 × 10^5^ primary neutrophil cells isolated from mouse bone marrow were placed into the insert of a Boyden chamber. The bottom compartment contained the RPMI medium with PBS or rmOPN at a dose of 10 μg/ml as a chemotactic stimulus. After 2 h, the migrated primary neutrophil cells were counted. A representative image of the migrated primary neutrophil cells labeled with PI (red fluorescence) on the bottom of the transwell membrane is shown. Cells were observed at × 200 original magnification. **(B)** Migrated primary neutrophils were counted in five random microscopic fields per well and averaged in each group. Data are expressed as means ± SEM (n = 4/group) and compared by Student’s *t* test (^*^ *P* <0.05 vs. PBS). Ab, antibody; OPN, osteopontin; PBS, phosphate-buffered saline; PI, propidium iodide; rmOPN, recombinant mouse OPN; SEM, standard error of the mean.

To examine the direct role of OPN for neutrophil migration in lungs, we injected rmOPN in lungs intratracheally and assessed neutrophil infiltration in lung tissues. As shown in Figure 6A and B, under normal condition the percentage of lung neutrophil content was negligible. However, administration of rmOPN into the lungs significantly increased the percentage of lung neutrophils as revealed by the Gr-1-positive cells. In order to validate our *in vivo* finding, we have carried out an *in vitro* experiment that also revealed rmOPN-mediated dramatic increase of primary neutrophil migration (Figure 6C and D). These data clearly demonstrate the role of OPN for neutrophil migration, *in vivo* and *in vitro*.

### rmOPN activates phosphorylation of FAK, ERK and p38 in neutrophils

**Figure 7:**
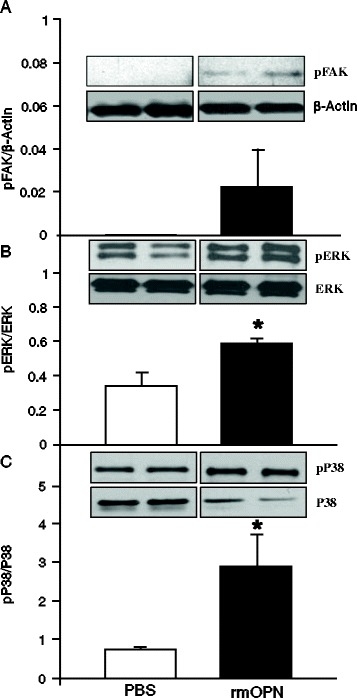
**rmOPN-mediated activation of FAK and MAP kinase signaling molecules in dHL-60 cells** ***.*** Differentiated HL-60 cells were incubated with PBS or rmOPN at a dose of 2 μg/mL for 90 min. **(A)** The status of phosphorylated FAK (pFAK) and β-actin, **(B)** pERK and total ERK and **(C)** pp38 and total p38 in each group was determined by Western blot. Blots were scanned and quantified with densitometry. Representative blots against these proteins are shown. Data are expressed as means ± SEM obtained from two independent experiments (n = 5/group) and compared by Students *t* test (*P <0.05 vs. PBS). ERK, extracellular signal-regulated protein kinase; FAK, focal adhesion kinase; MAP, mitogen-activated protein; OPN, osteopontin; PBS, phosphate-buffered saline; rmOPN, recombinant mouse OPN; SEM, standard error of the mean.

MAP kinases play pivotal roles in efficient neutrophil migration [34,35]. Integrins serve as the pivotal receptors for OPN-mediated signaling [11]. To reveal the mechanism of OPN-mediated neutrophil migration we used a human differentiated neutrophil cell line, dHL-60 cells. At first, to determine the integrin-mediated signaling after treatment with rmOPN in dHL-60 cells, phosporylation of the integrin-associated downstream molecule, FAK was examined. Neutrophils treated with rmOPN dramatically upregulated pFAK levels over that of controls, indicating it to be an essential molecule for OPN-mediated downstream signaling (Figure 7A). Furthermore, to assess the involvement of OPN in MAP kinase pathway, the phospholyration levels of ERK and p38 in the dHL-60 cells treated with rmOPN were determined by Western blot. In the dHL-60 cells pretreated by rmOPN, the phospholyration of these kinases were remarkably higher as compared with the PBS-treated dHL-60 cells, indicating OPN-mediated downstream signaling could be generated through FAK and MAP kinases (Figure 7B,C).

### rmOPN promotes neutrophil migration via ERK and p38 MAP kinase activation in vitro

**Figure 8:**
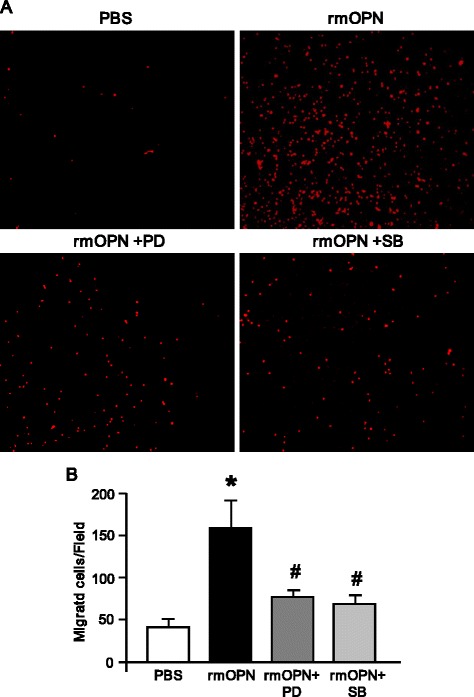
**Effect of rmOPN on dHL-60 cell migration** ***in vitro.*** Boyden chamber assay was performed as described in Materials and Methods. **(A)** A representative image of migrated dHL-60 cells labeled with PI (red fluorescence) on the bottom of the transwell membrane. Cells were observed at × 200 original magnification. **(B)** Migrated dHL-60 cells were counted in five random microscopic fields per well and averaged in each group. Data are expressed as means ± SEM (n = 4/group) and compared by one-way ANOVA and SNK method (^*^ *P* <0.05 vs. PBS; ^#^ *P* <0.05 vs. rmOPN). ANOVA, analysis of variance; OPN, osteopontin; PBS, phosphate-buffered saline; PI, propidium iodide; rmOPN, recombinant mouse OPN; SEM, standard error of the mean; SNK, Student-Newman-Keuls.

To examine the effect of rmOPN on neutrophil migration, dHL-60 cells were subjected to a Boyden chamber assay. In the PBS-pretreated dHL-60 cells, comparably higher numbers of migrated cells were detected at the bottom of the micropore membrane when rmOPN was used as a chemoattractant in the lower chamber (Figures 8A,B). However, pretreatment of the cells with ERK or p38 inhibitors significantly downregulated the dHL-60 cell migration towards rmOPN by 54% and 60%, respectively as compared with the PBS-treated dHL-60 cells (Figures 8A,B). Collectively, these data clearly revealed that the rmOPN-mediated dHL-60 cell migration occurred through the induction of the MAP kinase pathway.

## Discussion

Osteopontin plays diverse cellular functions in innate immune system starting from promoting T helper cell type 1 (Th1) skewing and extended through the differentiation and proliferation of immune reactive cells [31,36,37]. OPN also functions as a proinflammatory cytokine and recruits inflammatory cells to exaggerate tissue damage [11]. Considering its above features, we aim to reveal its expression, function and therapeutic implication in murine experimental sepsis. Our current study demonstrates an increased expression of OPN in lungs and spleen tissues, as well as in the plasma of septic animals. A strategy utilizing the neutralizing Ab against OPN dramatically reduced systemic inflammatory responses, organ injuries, and neutrophil infiltration into the lungs, leading to the improvement of the integrity of lung architecture and downregulation of MPO activity after sepsis. It is reasonable to think that the overall improvement of sepsis by neutralizing OPN could be due to the inhibition of systemic inflammatory and injury markers in general. Correspondingly, the decreased levels of MIP-2, a potent chemoattractant for neutrophils in plasma as well as in lungs after sepsis in animals treated with anti-OPN Ab might also indirectly regulate excess neutrophil migration at the infectious foci as well as in remote organs. Interestingly, our *in vivo* finding demonstrating excessive neutrophil migration in lungs as a result of intratracheal rmOPN injection provided direct involvement of OPN-mediated induction of ALI (Figure 6A,B), which might in turn be reversed by administrating neutralizing Ab against OPN to attenuate lung neutrophil migration in sepsis-induced ALI. Moreover, our *in vitro* approaches clearly confirmed OPN as a potent chemoattractant for neutrophil migration directly by upregulating MAP kinase pathway molecules, p38 and ERK with the fact that utilizing their inhibitors greatly diminished the OPN-mediated neutrophil migration. OPN can recognize several integrins, such as α_v_β_3_, α_v_β_5_, α_v_β_1_, α_8_β_1_ and others as its receptors for signal transduction [38]. Since these integrins are known to activate FAK [39-41], it is therefore meaningful to examine the effect of OPN for FAK activation in neutrophil cells.

OPN is strikingly upregulated at the sites of inflammation and during tissue remodeling [42,43]. As a proinflammatory cytokine, increased levels of OPN have been demonstrated in a variety of chronic inflammatory diseases including Crohn’s disease, cancer, atherosclerosis, aortic abdominal aneurysms, and autoimmune diseases [11-17]. In contrast, a few studies were performed to focus on its expression during acute inflammatory disease conditions. Studies using the human subjects, Vaschetto *et al.* have recently reported increased concentration of plasma OPN in sepsis patients [27]. In our study, we have also noticed significant upregulation of OPN in plasma as well as in lung and spleen tissues at 20 h after sepsis. Sepsis is characterized by a biphasic immunological phenomenon where the early acute hyperinflammatory phase is accompanied by the immunosuppressive phase at the late stage of sepsis [1]. In our current study, we have emphasized the early acute phase of sepsis to demonstrate the effects of anti-OPN Ab as a preventive approach. However, the setting up of an anti-OPN Ab-based treatment strategy in sepsis considering its pathophysiological window period in mice would reflect its clinical relevancy. Recently, Vaschetto *et al.* measured the levels of OPN for fifteen days in patients with high risk of developing septic shock and noticed increased levels of OPN started at four days before shock development [44]. Thus, OPN can be considered as the prognostic marker of shock and the therapeutic measures targeting OPN should be implemented based on this window period. However, a recent article strongly suggests that the mouse models to study sepsis differ from what is seen clinically in humans [45]. This is due to the fact that rodents are highly resistant to most types of induced inflammation compared with humans. Moreover, compared with what is seen in sepsis syndrome in humans, mice within a CLP model tend to have a shorter duration of the disease, which is often terminated with a quite sudden death and less organ failure [45]. As a result of the discrepancies between human and animal models of sepsis, the actual window period of the disease can vary and the treatment strategy may not be truly transferable to the clinical practice. Considering the aberrant expression of OPN in lungs, we next attempted to delineate whether or not it plays a deleterious role in lungs during sepsis. OPN’s role as a chemoattractant to promote the migration of immune cells to the site of inflammation has been demonstrated in earlier studies [11,23]. Nonetheless, the majority of the reports were mainly based on the role of OPN on macrophage migration. Correspondingly, Bruemmer *et al.* also have shown that acute macrophage infiltration was dramatically diminished in OPN-null mice compared to wild-type mice in a thioglycollate-induced peritonitis model [46]. In our current study, we for the first time revealed the role of OPN for neutrophil migration into the lungs during polymicrobial sepsis in mice. Consistent with our findings, OPN was shown to be upregulated and associated with neutrophil and macrophage infiltration in glioblastoma, the most invasive type of brain tumors/glioma [47]. In our study, administration of the neutralizing Ab against OPN in mice during sepsis greatly ameliorated the contents of infiltrating neutrophils in the lungs, thereby protecting mice from developing ALI induced by sepsis. Similar to our approach, a recent study has also shown that the antibody-mediated neutralization of OPN significantly reduced the obesity-induced inflammation and insulin resistance in mice [48]. Beside this report, Fortis *et al.* have recently demonstrated reduced levels of plasma cytokines and chemokines in OPN knockout mice in a murine model of sepsis [49], which indirectly validated our approach for attenuating sepsis-associated ALI in mice by the treatment of anti-OPN Ab. Although the above study revealed a deleterious role of OPN in sepsis, the mechanism remained unexplored. In our current study, we therefore not only demonstrated the beneficial outcomes of OPN-neutralizing Ab in sepsis-induced ALI, but also elucidated intracellular signaling events involving the activation of ERK and P38 MAP kinases to govern OPN-induced neutrophil migration. Hence, our approach for utilizing anti-OPN Ab as a potential therapeutic tool for treating ALI caused by excessive neutrophil infiltration is permissible. It has previously been demonstrated that the MAP kinases play a pivotal role in cell migration [34]. The involvements of ERK and P38 MAP kinases for neutrophil migration have been well characterized in recent studies [50,51]. In contrast, the stress-activated protein kinases (SAPK)/Jun amino-terminal kinases (JNK) are best known for their pivotal roles in cellular growth, differentiation, survival, and apoptosis [52] and are less focused on neutrophil migration. We therefore emphasized evaluating the phosphorylation status of ERK and P38 for OPN-mediated neutrophil migration. Beside this, FAK and its signaling pathways are also involved in cell migration [53]. In an *in vitro* system we have noticed considerable upregulation of ERK, p38 and FAK in human neutrophil cell line treated with rmOPN. Besides, we also confirmed the involvement of ERK and p38 for OPN-mediated neutrophil migration by using their inhibitors, which showed dramatic decrease in OPN-mediated neutrophil migration. Moreover, our study showing remarkable upregulation of pFAK by rmOPN treatment not only indicated the pFAK’s role in cellular migration, but also clarified its upstream signaling event that could be mediated through the interaction of its N-terminal RGD motif and α_v_β_3_-integrin. Recently, a bidirectional regulatory role of MAP kinase for neutrophil migration has been demonstrated where the activation of p38 molecule enhanced the chemokine-mediated neutrophil migration, while the activation of ERK pathway inhibited their migration [50]. In this study, they have utilized a potent chemokine, fMLP, to show the bidirectional regulatory roles of MAP kinases that mediated through the G-protein coupled receptor kinase-2 (GRK-2). On the other hand, we have utilized a different chemoattractive molecule, OPN, which could promote the downstream signaling for neutrophil migration via recognizing its integrin receptor without involving the GRKs.

Apart from the direct neutrophil infiltration in lungs, ALI can also be aggravated by elevated systemic/local proinflammatory cytokines and chemokines, which may indirectly promote immune cell infiltration in lungs. Utilizing the OPN knockout mice Van der Windt *et al.* demonstrated reduced plasma levels of proinflammatory cytokines and chemokines in *Streptococcus pneumonia*-infected animals [24]. Similarly, in our study utilizing the OPN blocking strategy by its neutralizing Ab significantly attenuated the IL-6, IL-1β and MIP-2 levels not only in the blood but also in lungs, which we suggest that the anti-OPN Ab-mediated mitigation of ALI could also be due to the downregulation of proinflammatory cytokines and chemokines. Likewise, neutralizing OPN may generate potential beneficial outcomes in an array of inflammatory diseases.

## Conclusions

In conclusion, we have clearly demonstrated the protective role of anti-OPN Ab against ALI after sepsis in mice by inhibiting neutrophil migration in lungs. In addition, we have also revealed the intracellular signaling cascade for neutrophil migration activated by osteopontin through the upregulation of MAP kinase pathway, which may further contribute to novel drug development for inhibiting neutrophil migration in sepsis-induced ALI. Further studies to delineate effective dose and timing of administration will be helpful for future clinical trials with ALI patients, which not only save their lives, but also relieve economic burden.

## Key messages

- OPN expression is upregulated in plasma and multiple organs (lungs and spleen) after sepsis in mice.
- OPN neutralization decreases systemic levels of proinflammatory cytokines as well as organ injury markers after sepsis in mice.
- OPN neutralization improves the integrity of lung architecture and attenuates neutrophil infiltration as well as MPO activity in the lungs after sepsis in mice, resulting in the protection against sepsis-induced ALI.
- OPN promotes neutrophil migration via ERK and p38 MAP kinase activation.

## Abbreviations

Ab: antibody
ALI: acute lung injury
ALT: alanine aminotransferase
ARDS: acute respiratory distress syndrome
AST: aspartate aminotransferase
BSP-I: bone sialoprotein-I
CLP: cecal ligation and puncture
DC: dendritic cells
DMSO: dimethyl sulfoxide
ELISA: enzyme-linked immunosorbent assay
ERK: extracellular signal-regulated protein kinase
ETA-1: early T lymphocyte activation
FAK: focal adhesion kinase
FBS: fetal bovine serum
GRK: G protein-coupled receptor kinase
H&E: hematoxylin and eosin
ICU: intensive care unit
IgG: immunoglobulin G
IL: interleukin
JNK: Jun N-terminal kinase
kDa: kiloDalton
LDH: lactate dehydrogenase
MAP: mitogen-activated protein
MIP: macrophage inflammatory protein
MMP: matrix metalloproteinase
MOF: multiple organ dysfunction
MPO: myeloperoxidase
NK: natural killer
OD: optical density
OPN: osteopontin
PBS: phosphate-buffered saline
PCR: polymerase chain reaction
PI: propidium iodide
rmOPN: recombinant mouse OPN
ROS: reactive oxygen species
SAPK: stress-activated protein kinase
SEM: standard error of the mean
SPP-1: secreted phosphoprotein-1
TBST: Tris-buffered saline with Tween-20
Th1: T helper type 1
